# UV-Curable L(-)–Borneol-Functionalized Antibacterial Hydrogels for Packaging of Fresh-Cut Banana and Cherry Tomato

**DOI:** 10.3390/gels12050381

**Published:** 2026-04-30

**Authors:** Jizhong Yuan, Yaohuang Jiang, Mengle Liu, Peipei Wu, Guoxian Feng, Yanchun Yu, Xiongfa Yang

**Affiliations:** 1 Key Laboratory of Organosilicon Chemistry and Material Technology, Ministry of Education, Zhejiang Key Laboratory of Organosilicon Material Technology, College of Material, Chemistry and Chemical Engineering, Hangzhou Normal University, Hangzhou 311121, China; 2023112009034@stu.hznu.edu.cn (J.Y.); 2024112009017@stu.hznu.edu.cn (M.L.); 2024112009066@stu.hznu.edu.cn (P.W.); 2024111009098@stu.hznu.edu.cn (G.F.); 2 College of Life and Environmental Sciences, Hangzhou Normal University, Hangzhou 311121, China; 20263007@hznu.edu.cn

**Keywords:** UV-curable hydrogel, antibacterial, L(-)–borneol, fresh-cut fruits and vegetables

## Abstract

UV-curable L(-)–borneol-functionalized antibacterial hydrogels for packaging fresh-cut banana and cherry tomato (UV-LBs) were designed from L(-)–borneol-functionalized polyurethane acrylate prepolymers (LB-PUAs) and thiol-functionalized PVA (PVA-SH) using a thiol-ene click reaction initiated by UV light. UV-LBs exhibit unique properties, including excellent thermal stability, high mechanical performance and quite high antibacterial efficiency. The initial thermal decomposition temperature (T*_d5_*), tensile strength and elongation at break are in the range of 225–240 °C, 1.38–2.05 MPa and 44.4–68.6%, respectively. The antibacterial efficiency of UV-LBs against *Staphylococcus aureus* (*S. aureus*), *Escherichia coli* (*E. coli*), and *Monilia albican* (*M. albican*) can reach 67.4%, 75.6% and 83.7%, respectively. The storage time of packaged fresh-cut banana and cherry tomato can be extended from 12 h to 30 h and 4 d to 5 d, respectively.

## 1. Introduction

Fruits and vegetables are important foods to human beings because they are rich in vitamins, minerals and dietary fiber [1]. Nevertheless, they are susceptible to spoilage due to microbial infections during the course of preservation and transportation [1,2,3]. As reported, about 42% of fruits and vegetables are wasted during the course of preservation and transportation every year [1,4]. Recently, due to the faster and faster pace of modern life, the demand for convenient ready-to-eat foods such as fresh-cut fruits and vegetables is growing [5,6]. However, the cutting operations can induce mechanical injury, which will inevitably reduce their storage time as a result of bacterial infection [7,8]. Obviously, developing packaging materials to prolong the preservation time is extremely urgent. The preservation methods for fruits and vegetables, including packaging with coatings [9], adding antioxidant agents [10], and storing under air conditioning [11], have major drawbacks, including high costs, the requirement for specialized equipment and technology, and a great possibility of spoiling the flavor [12]. So, developing antibacterial packaging materials for the preservation of fresh fruits and vegetables is an urgent need [1,13,14].

Antibacterial hydrogels, soft, three-dimensional (3D) materials cross-linked by reversible covalent bonds or physical molecular interactions [15], possessing unique performance characteristics of hydrophilicity and antimicrobial activity against bacteria, have gained increasing attention in the food industry [16,17]. The antibacterial films prepared with polyvinyl alcohol (PVA) and nitrogen-doped carbon dots can delay the browning of fresh-cut apples [18]. Incorporating purple sweet potato anthocyanin and silver nanoparticles into antibacterial composite films of chitosan (CS)/PVA can extend the storage time of strawberries to 13 d at 4 °C [19]. By introducing Cu-tannic acid nanoparticles into a CS-gelatin matrix, the films exhibit killing efficiency against *E. coli* and *S. aureus* of over 99% and double the strawberries’ storage time [20]. Antibacterial polysaccharide-based films prepared from sodium alginate and persimmon polysaccharide extractions incorporating silver nanoparticles can maintain the stability of pH and vitamin C during the storage period [21]. However, these nanoparticle-containing antibacterial hydrogels have the shortcoming of toxicity [22].

In response to these challenges, many antibacterial hydrogels were designed without toxic nanoparticles. Stancu A. I. et al. developed biocompatible CS-based antibacterial hydrogels with antibiofilm effects of 2.34 mg/mL for pharmaceutical application from natural complexes, such as tinctures of *Verbena officinalis*, *Aloysia triphylla*, *Laurus nobilis* essential oil, and a beta-cyclodextrin-clove essential oil [23]. Galaburri G. et al. prepared antibacterial materials from starch or polysaccharides in combination with a hydrochloride polymer of linear polyethyleneimine, which showed high antibacterial efficiency against *S. aureus* [24]. Somwongin S. et al. developed antibacterial hydrogels for topical anti-acne treatment using propolis, honey and royal jelly. They revealed that the gels prepared with 1% *w*/*w* propolis showed the strongest antibacterial activity, with an inhibition zone of about 20.0 mm against *S. aureus* [25]. Pectin-based antibacterial films functionalized with Schiff base groups displayed fairly high inhibition against *E. coli*, *S. aureus*, and *B. cinerea* over 24–48 h, and papaya packaged with these films can maintain optimum firmness over 10 d [1]. Eco-friendly coatings fabricated from sodium carboxymethyl cellulose, lipopeptides and gelatin can extend the storage time of blueberries by 12.5% [26]. Packaging films developed from soy protein isolate, gelatin, rosemary-modified bentonite and glycerol and used for fresh lemon slice preservation can reduce weight loss by about 37.9% and maintain freshness from 2 d to 4 d [27]. Antibacterial carrageenan-based films prepared with melanin extract can extend the storage time of strawberries to 5 d at 25 ± 2 °C [28]. PVA, a cost-effective, biodegradable and biocompatible polymer, is an ideal raw material for preparing hydrogels for food preservation [29]. A hydrogel film prepared from PVA, sodium carboxymethyl cellulose, ginkgo biloba leaf extract and tannic acid can prolong the storage time of strawberries by 7 d [30]. An antibacterial hydrogel prepared from PVA, CS, soy hull nanocellulose and anthocyanin displayed an antibacterial efficiency of about 90.6%, which can prolong the storage time of salmon meat from 6 d to 14 d [31]. Gong et al. embedded a gallic acid-phycocyanin fiber mesh hydrogel into a PVA to produce an antibacterial fruit-maintaining film, and the hydrogels can prolong the storage time of grapes and blueberries to 13 d and 20 d at ambient temperature, respectively [32].

It is widely known that hydrogels prepared via physical molecular interactions often suffer from low mechanical properties and poor thermal stability [15,33]. To address these shortcomings, some hydrogels were cross-linked by Zn^2+^ or Fe^2+^ [13,34,35]. Some other hydrogels were cross-linked by covalent bonds using a cross-linking agent [36]. For example, Wang et al. used CS, PVA and ε-PL to prepare a cross-linked multifunctional hydrogel, and the hydrogel can extend the storage time of chilled chicken to 8 d [37]. Similarly, organic cross-linking agents such as 1-ethyl-3-(3-dimethylaminopropyl) carbodiimide and N-hydroxysuccinimide were utilized to form stable amide covalent bonds to improve the mechanical property of hydrogels [38]. However, the network structure of these hydrogels will consist of either organic or inorganic compounds, which will limit their further application in the food industry [39].

Increasing attention has been paid to UV curing technology due to its distinctive merits, including eco-friendliness, energy saving and high efficiency [16]. Polyurethanes (PUs) are among the most employed high-performance polymer materials in fields, such as aerospace, automotive, electronics, and deep-sea exploration, due to their excellent mechanical properties, biocompatibility, chemical stability, and flexibility [40]. As reported, UV-curable pH-sensitive antibacterial hydrogels for monitoring the freshness of beef can be prepared from choline chloride and bromophenol red-functionalized CS and thiol-functionalized PU via a thiol-ene click reaction initiated by UV light [16]. Antibacterial and pH-sensitive smart UV-curable hydrogels for shelflife extension of chicken can also be developed from eugenol and bromocresol green-functionalized PU and thiol-modified CS [17]. These works reveal that UV curing technology can be adopted to develop high-performance hydrogels.

Borneol, a natural and lipophilic Chinese herbal medicine extracted from a variety of medicinal plants, is an ideal less toxic, environmentally friendly and broad-spectrum antibacterial material [41,42]. Zhao et al. designed natural antibacterial hydrogels via the Schiff base cross-linking of dialdehyde dextran-grafted borneol and carboxymethyl CS. Thanks to the cooperation of borneol groups and positively charged ammonium ion groups, the hydrogels can inhibit the growth of *E. coli* and *S. aureus* in 24 h, and they also demonstrate fairly good in vitro cytocompatibility [43]. Gao et al. developed antibacterial hydrogel with Yunnan Baiyao and borneol, which demonstrated outstanding stretch ability, with elongation at break of ~1876%, and an inhibition zone of 8 mm against *S. aureus* [44]. Hydrogels for infected burn wound healing were prepared with gallic acid-functionalized PVA, 3-aminobenzeneboronic acid-functionalized sodium alginate, Zn^2+^, and CS-coated borneol nanoparticles [45]. Inspired by these interesting works, antibacterial UV-curable antibacterial hydrogels were designed from L(-)–borneol-functionalized PU acrylate prepolymers (LB-PUAs) and thiol-functionalized PVA (PVA-SH) via a UV-initiated thiol-ene click reaction. The antibacterial performance, thermal stability and mechanical performance were investigated. The preservation investigation reveals that the hydrogels can obviously extend the storage time of fresh-cut banana and cherry tomato. It provides a potential strategy to develop high-efficiency antibacterial hydrogels for packaging the fresh-cut fruits and vegetables with natural Chinese herbal medicine-based polymers.

## 2. Results and Discussion

### 2.1. Characterization of FT-IR

**Figure 1:**
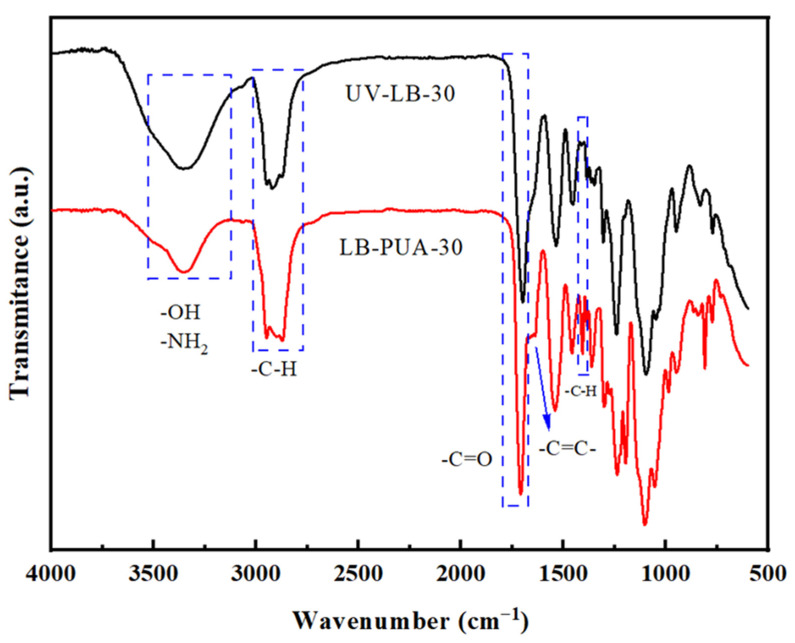
FT-IR spectra of LB-PUA-30 and UV-LB-30.

LB-PUA-30 and UV-LB-30 were characterized by FT-IR analysis, shown in Figure 1. The absorption in the range of 3458–3210 cm^−1^ is assigned to the stretching vibrations of –OH and –NH_2_, while that in the range of 2956–2870 cm^−1^ is assigned to the stretching vibration of –C–H. The absorptions at 1699–1703 cm^−1^ and 1418–1420 cm^−1^ are assigned to the stretching vibration of –C=O and deformation vibration of –CH_3_ in the –NHCOCH_3_ groups, respectively. There is weak characteristic absorption at 1620 cm^−1^ assigned to –C=C– in acrylate groups in the FT-IR spectrum of LB-PUA-30, while there is no corresponding characteristic absorption of –C=C– in the spectrum of UV-LB-30, which indicates that UV-LBs can be cured almost completely for 20 s each face.

### 2.2. Thermal Stability Investigation of UV-LBs

**Figure 2:**
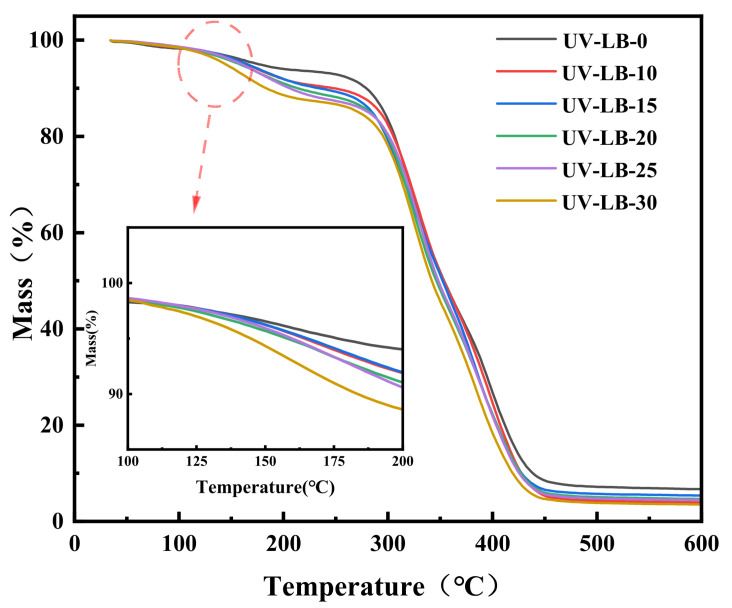
TGA curves of UV-LBs.

Thermal stability has a key impact on the application of antibacterial hydrogels in the packaging of food cooked at relatively high temperatures or medical materials requiring high-temperature sterilization. So, TGA analysis of UV-LBs was studied. As presented in Figure 2, T_d5_ of UV-LBs decreased from 225 °C to 240 °C with the increment of the molar ratio of L(-)–borneol to HPA from 30:70 to 0:100 due to the poor thermal stability of L(-)–borneol groups [46]. Above all, the hydrogels prepared have fairly good thermal stability.

### 2.3. Swelling Behavior of UV-LBs

**Figure 3:**
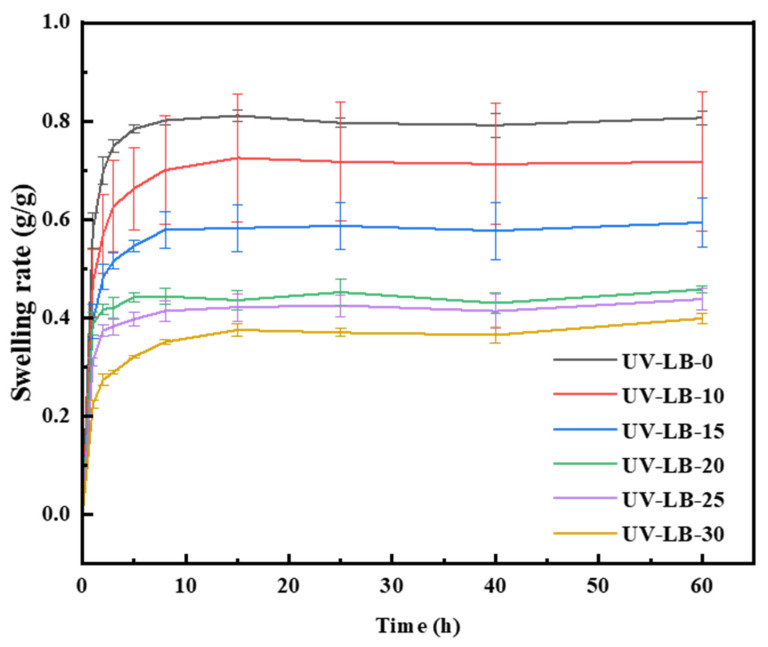
Variation of swelling rate with time.

The swelling rate has a significant impact on the water absorption ability and stability of hydrogels, which is essential for practical applications in food packaging, wound healing and wearable electronics [16,47,48]. Therefore, the swelling rate of UV-LBs prepared with different molar ratios of L(-)–borneol to HPA was evaluated (Figure 3). Clearly, the swelling equilibrium was established after the UV-LB samples were immersed for 10 h in deionized water, and the swelling rate is in the order of UV-LB-30 < UV-LB-25 < UV-LB-20 < UV-LB-15 < UV-LB-10 < UV-LB-0. When the molar ratio of L(-)–borneol to HPA was raised from 0:100 to 30:70, the swelling rate of UV-LBs was reduced from 80% to 32%, respectively, which means the water absorption ability decreased because the amount of L(-)–borneol groups grafted increased.

### 2.4. DMA Analysis of UV-LBs

**Figure 4:**
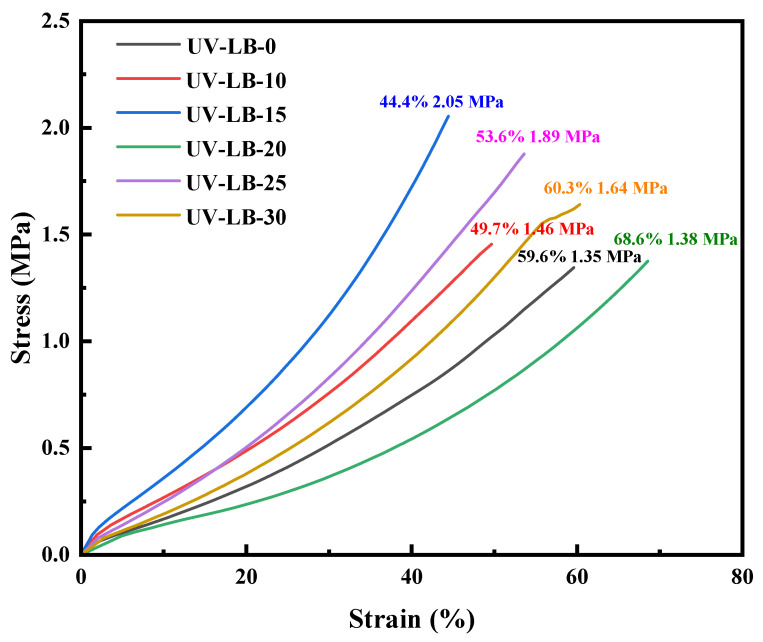
Strain–stress curves of hydrogel films measured by DMA.

The mechanical performance of UV-LBs was evaluated through DMA analysis, shown in Figure 4. The tensile strength and elongation at break of UV-LBs are in the range of 1.38–2.05 MPa and 44.4–68.6%, respectively. As we know, one efficient chemical modification strategy is the incorporation of rigid chemical groups to improve the mechanical performance of materials [49,50]. Therefore, when the molar ratio of L(-)–borneol to HPA is 15:85, the highest tensile strength of UV-LBs (2.05 MPa) is achieved by balancing the flexible units and the rigid units [51]. Making a comparison with the non-ionic UV-curable hydrogel prepared by Liu with a tensile strength of 0.1–0.43 MPa, it is shown that the UV-LBs have higher tensile strength [51].

### 2.5. Antibacterial Property of UV-LBs

#### 2.5.1. Antibacterial Property Evaluated with the Coated Plates and Colony Count Method

**Figure 5:**
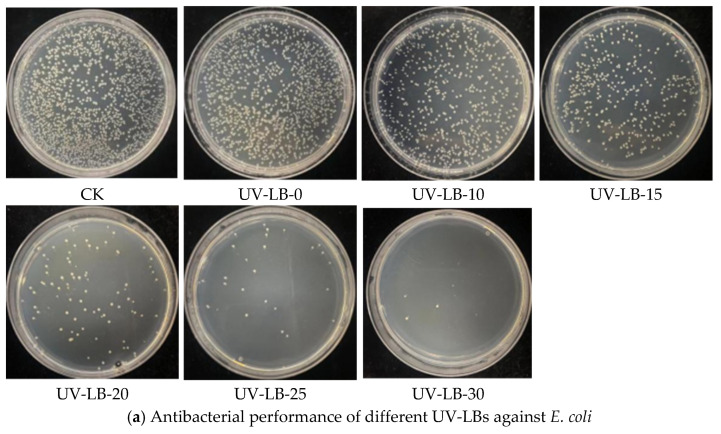

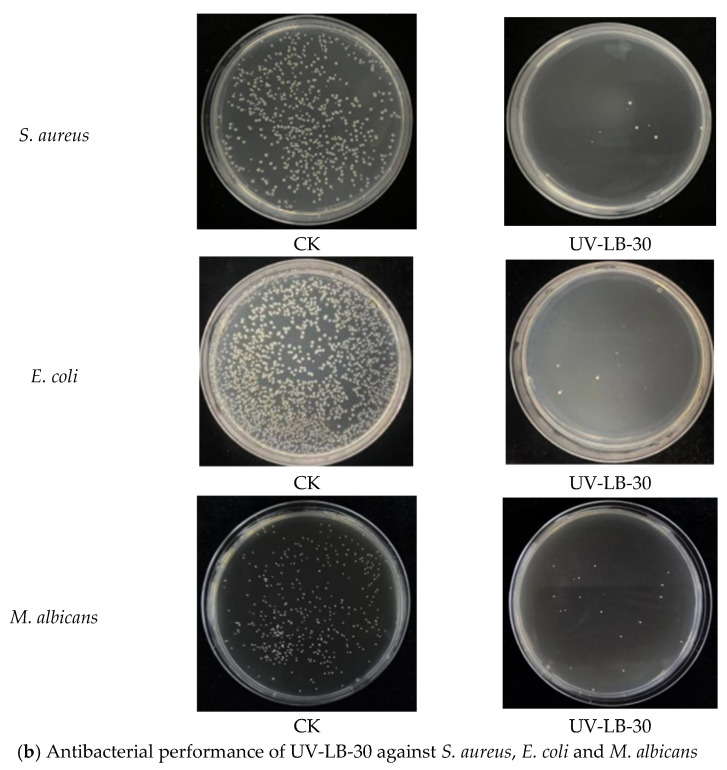
Antibacterial property evaluated with the coated plates and colony count method.

The antibacterial property of UV-LBs was studied using the coated plates and colony count method, as shown in Figure 5. From Figure 5a, it can be concluded that the antibacterial efficiency against *E. coli* increased with an increasing molar ratio of L(-)–borneol to HPA because the number of bacterial colonies was correspondingly reduced. It reveals that a higher amount of L(-)–borneol groups in UV-LBs will achieve higher antibacterial efficiency. As shown in Figure 5b, the antibacterial efficiency against *S. aureus, E. coli* and *M. albicans* was 67.4%, 75.6% and 83.7%, respectively. So, the UV-LBs exhibit quite high antibacterial efficiency, particularly for *M. albicans*.

#### 2.5.2. OD_600_ Evaluation of UV-LBs Using Co-Culturing Method

**Figure 6:**
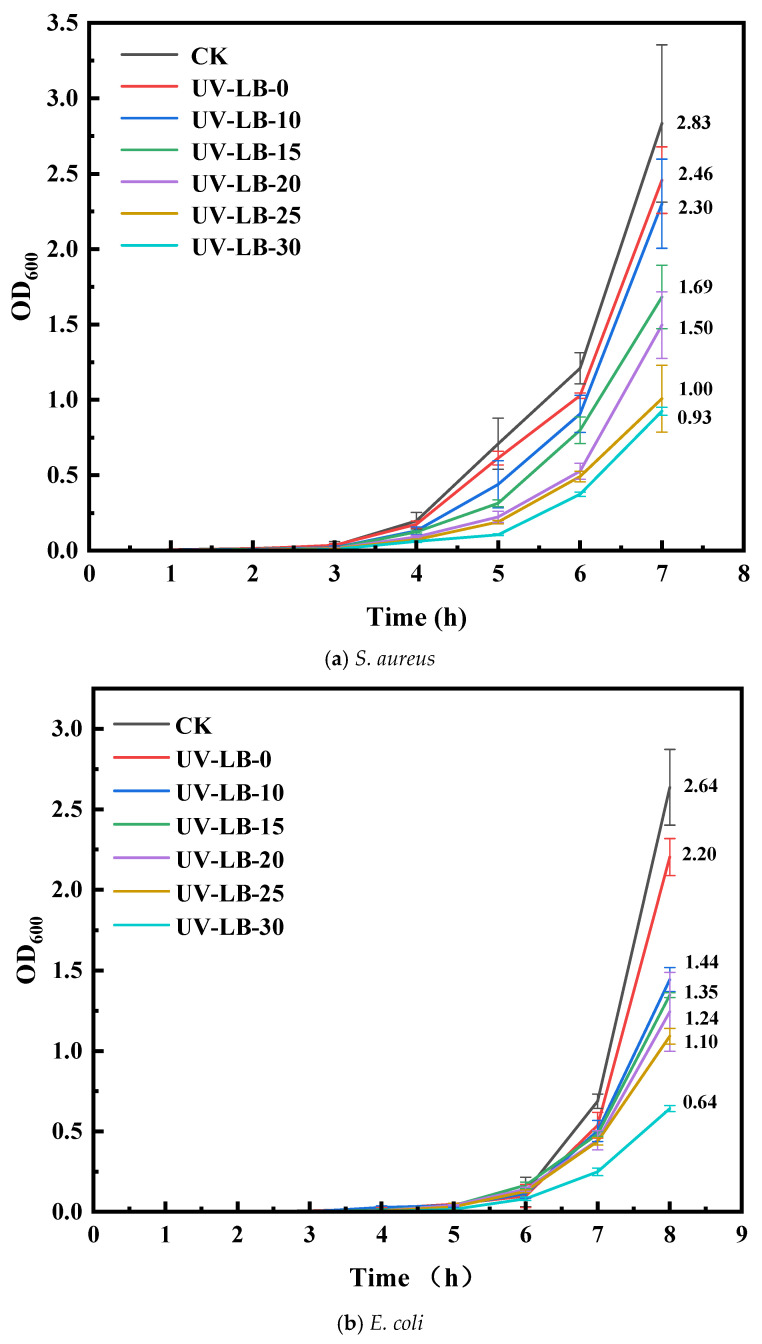

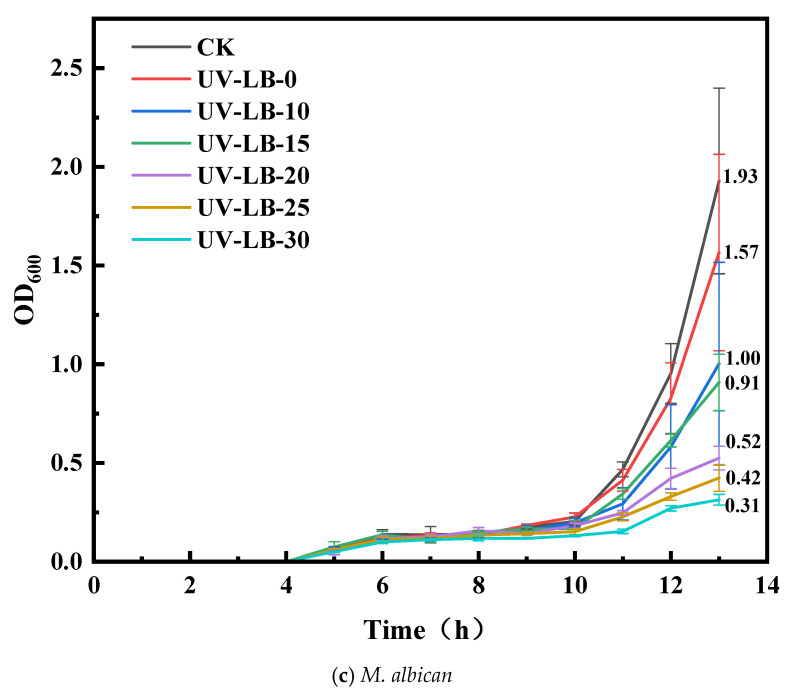
OD_600_ value measured with co-culturing method.

To estimate the antibacterial efficiency using the co-culturing method, the OD_600_ value was measured, as shown in Figure 6. Under the same condition, a lower OD_600_ value usually means higher antibacterial efficiency [16,17]. The OD_600_ values of UV-LBs were in the sequence of UV-LB-30 < UV-LB-25 < UV-LB-20 < UV-LB-15 < UV-LB-10 < UV-LB-0 < CK, which implies antibacterial efficiency of UV-LBs against *S. aureus*, *E. coli* and *M. albican* in the order of CK < UV-LB-0 < UV-LB-10 < UV-LB-15 < UV-LB-20 < UV-LB-25 < UV-LB-30. So, a higher molar ratio of L(-)–borneol to HPA will achieve higher antibacterial efficiency due to a higher content of L(-)–borneol groups. Hence, UV-LB-30 exhibited the highest antibacterial efficiency of 67.4% after 7 h against *S. aureus*, 75.6% after 8 h against *E. coli*, and 83.7% after 13 h against *M. albicans*.

#### 2.5.3. Bacterium and Fungi Morphology Study

**Figure 7:**
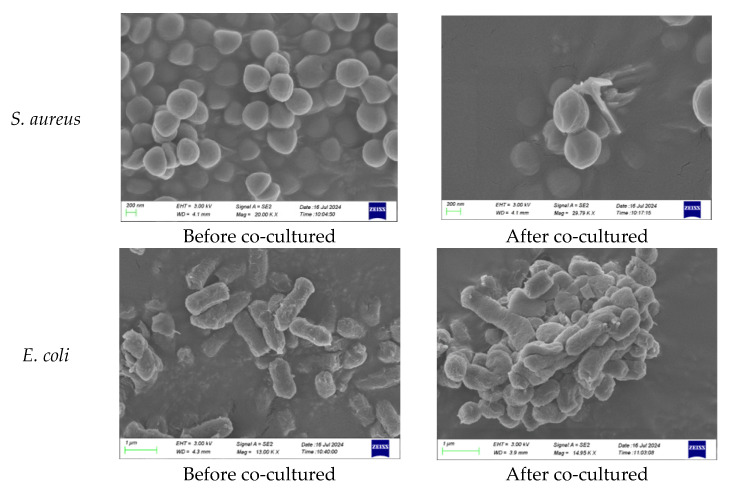

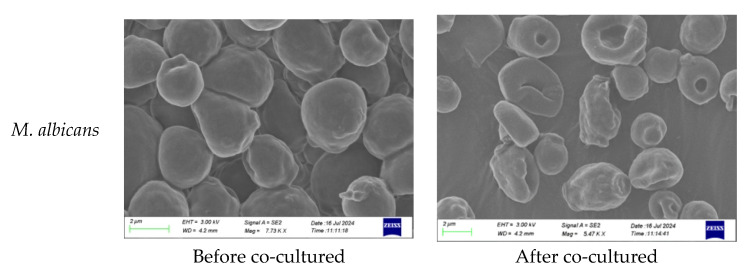
Cell morphology of *S. aureus*, *E. coli* and *M. albican* before and after co-culture.

The morphology of *S. aureus*, *E. coli* and *M. albican* co-cultured with UV-LB-30 was investigated using scanning electron microscope (SEM) analysis, shown in Figure 7. Clearly, the cell morphology of these bacteria and fungi after 24 h co-cultivation was either deformed or cracked, which means the bacteria and fungi were killed by the attachment of UV-LBs.

### 2.6. Cytotoxicity Assay

**Figure 8:**
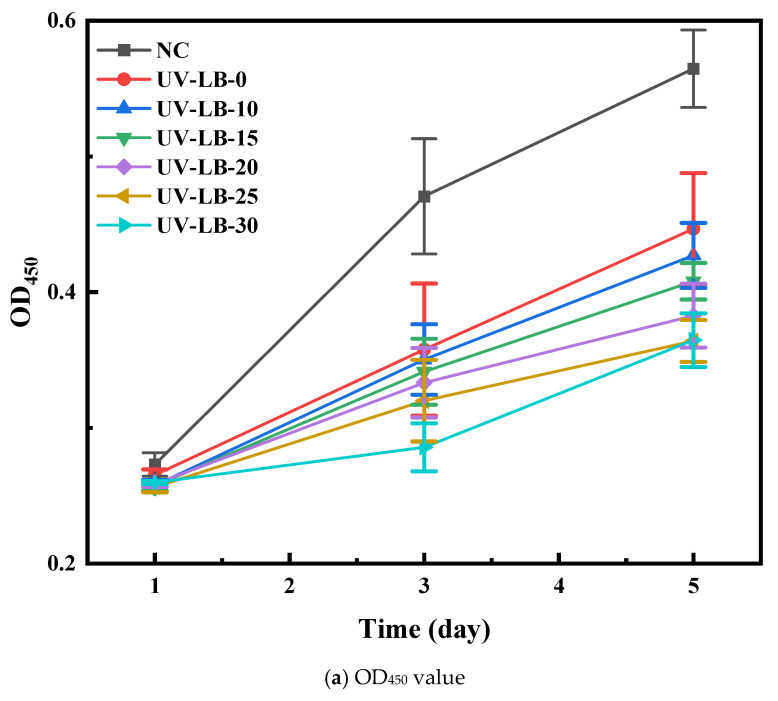

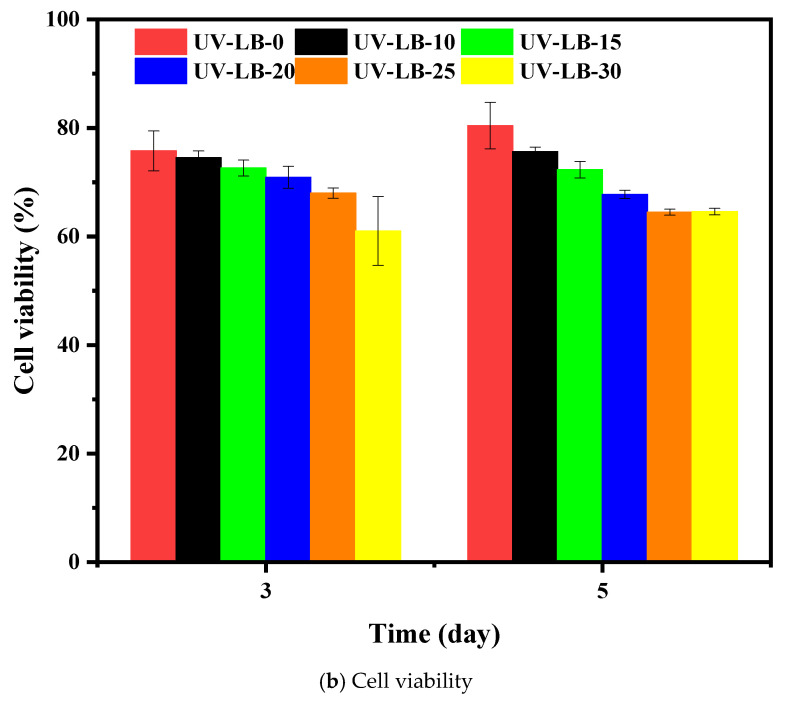
Cytotoxicity study of UV-LBs against L929 cells.

OD_450_ values and cell viability of UV-LBs against L929 cells were studied. As presented in Figure 8a, after 5 d, the OD_450_ values of UV-LB-grafted L(-)–borneol groups were in the range of 0.35–0.49, which were lower than that of UV-EB-0 (NC, about 0.56). As shown in Figure 8b, the cell viability of UV-LB-grafted L(-)–borneol groups was in the range of 60–80%, which suggested the UV-LB-grafted L(-)–borneol groups had fairly low toxicity. Though the toxicity increased with an increase in L(-)–borneol groups grafted, it is lower than borneol, a natural, less toxic, environmentally friendly and broad-spectrum antibacterial material [41,42].

### 2.7. Packaging Experiment of Fresh-Cut Banana and Cherry Tomato

**Figure 9:**
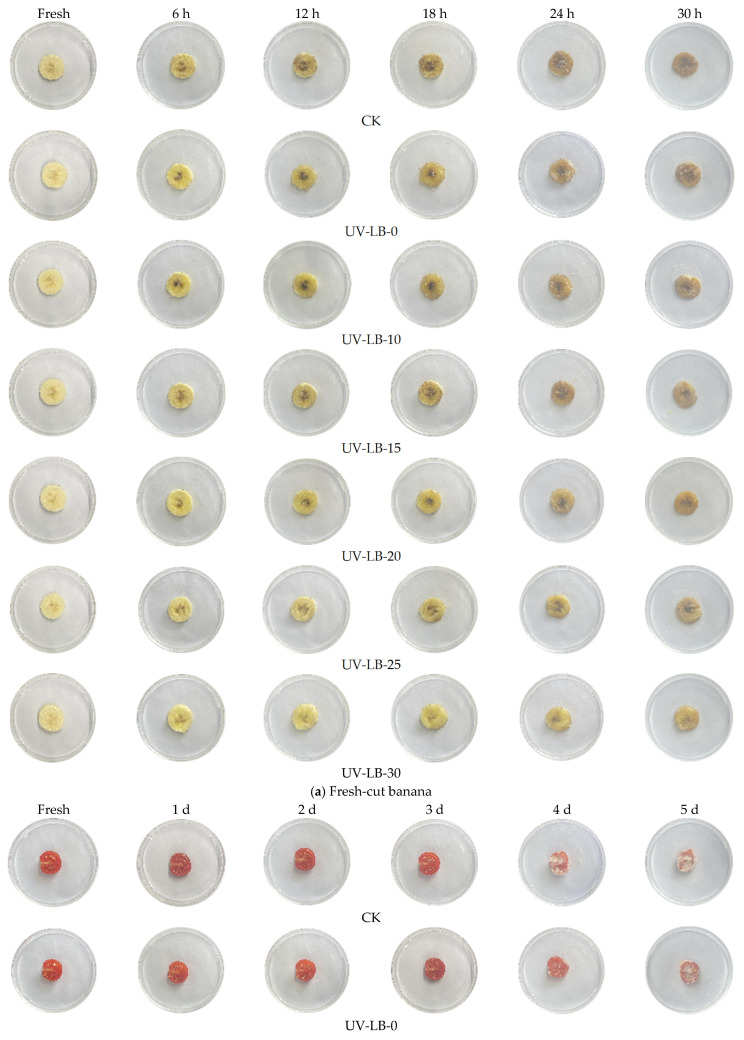

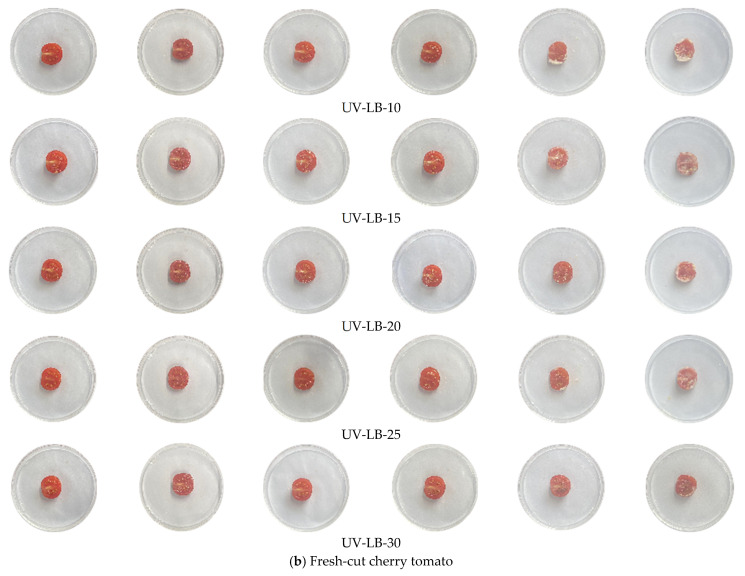
Packaging investigation.

The preservation of fresh-cut fruits and vegetables is quite difficult because of the mechanical injury induced by the cutting process, which makes them susceptible to bacteria [7,8]. As depicted in Figure 9, a packaging investigation of fresh-cut cherry tomato and banana by UV-LBs was carried out. As demonstrated by Figure 9a, the banana slice of CK showed some black spots of spoilage after 6 h, and it was clearly spoiled after 18 h. As for the fresh-cut banana slice packaged by UV-LB-10, though there were some spoiled black spots after 6 h, the content of spoilage was much less than that of CK. After being packaged for 24 h, the fresh-cut banana slice packaged by UV-LB-10 began to clearly deteriorate. When it comes to the fresh-cut banana slice packaged by UV-LB-25, the spoiled black spots occurred after being packaged for 18 h, and slight deterioration occurred after being packaged for 30 h. Clearly, when the fresh-cut banana slice was packaged by UV-LB-30, there were almost no black deterioration spots occurring after being packaged for 24 h. Even with those packaged by UV-LB-30 for 30 h, there was almost no sign of deterioration. So, it can be said that the storage time of fresh-cut bananas packaged by UV-LB-30 can be extended from 12 h to 30 h.

As demonstrated by Figure 9b, the CK, the fresh-cut cherry tomatoes packaged by UV-LB-0 and UV-LB-10, showed obvious deterioration after 4 d, while the fresh-cut cherry tomatoes packaged by UV-LB-15, UV-LB-20 and UV-LB-25 began to deteriorate clearly after being packaged for 5 d. Clearly, the sample packaged by UV-LB-30 showed only slight deterioration after being packaged for 5 d. This means that the storage time of fresh-cut cherry tomato can be extended from 4 d to 5 d through the packaging of UV-LB-30.

Overall, from the packaging experiment of both fresh-cut banana and cherry tomato above, it can be concluded that the storage time is extended, attributed to the increment in the amount of L(-)–borneol groups grafted on UV-LBs.

## 3. Conclusions

Antibacterial UV-curable antibacterial hydrogels were designed from LB-PUAs and PVA-SH through a UV-initiated thiol-ene click reaction. The T*_d_*_5_ of UV-LBs ranges from 225 °C to 240 °C, which decreases with an increment in the molar ratio of L(-)–borneol to HPA from 30:70 to 0:100. UV-LBs demonstrate good mechanical performance because their tensile strength and elongation at break of UV-LBs are in the range of 1.38–2.05 MPa and 44.4–68.6%, respectively. When the molar ratio of L(-)–borneol to HPA is 15:85, the UV-LB-15 obtained reaches the highest tensile strength of 2.05 MPa by balancing the flexible units and the rigid units. A higher content of L(-)–borneol groups will lead to higher antibacterial efficiency. The UV-LB-30 demonstrates the highest antibacterial efficiency of 67.4% after 7 h against *S. aureus*, 75.6% after 8 h against *E. coli*, and 83.7% after 13 h against *M. albicans*. The storage time of fresh-cut banana and cherry tomato packaged by UV-LB-30 can be extended from 12 h to 30 h and 4 d to 5 d, respectively. It provides a potential strategy to prepare antibacterial hydrogels for the packaging of foods with natural Chinese herbal medicine-based polymers.

## 4. Materials and Methods

### 4.1. Materials and Reagents

L(-)–borneol (98%), hydroxypropyl acrylate (HPA) and poly(ethylene glycol) (PEG 600, A. R., average molecular weight is about 600) were bought from Macklin Biochemical Co., Ltd. (Shanghai, China). Trimethylolpropane (TMP, 98%) and poly(vinyl alcohol) (PVA, 1799) were from Aladdin Biochemical Technology Co., Ltd. (Shanghai, China). Acetic acid glacial (A. R.) and 3–mercaptopropionic acid were purchased from Meryer (Shanghai, China) Chemical Technology Co., Ltd. 2–Hydroxy–2– methyl–1–phenylacetone (Irgacure–1173, 99.0%), ditin butyl dilaurate (A. R.), absolute ethanol, concentrated sulfuric acid (98%) and isophorone diisocyanate (IPDI, 98%) were purchased from Sinopharm Chemical Reagent Co. Ltd. *S. aureus*, *E. coli* (ATCC 25922), *M. albican* and L929 cells were supplied by Ningbo Taisituo Biotechnology Co., Ltd.

### 4.2. Synthesis of Thiol-Functionalized PVA (PVA-SH)

Typically, 20.0 mL acetic acid glacial, 36.0 mL acetic anhydride, 0.40 mL concentrated sulfuric acid and 40.0 mL 3-mercaptopropionic acid were introduced into a 500 mL round-bottom flask in turn and mixed thoroughly. Then, an aqueous solution of 4.00 g PVA in 100 mL deionized water was added, and the reaction was conducted at room temperature for 24 h. After that, 200 mL absolute ethanol was added, and a white floc was produced. Finally, white solid PVA-SH was obtained after the floc was centrifuged for 10 min at 1500 r/min, washed with absolute ethanol at least 3 times, put into a vacuum oven at 60 °C and dried overnight. After 7.0 g PVA-SH was dissolved completely in 100 mL deionized water, the PVA-SH aqueous solution was produced and kept in a single-necked bottle with a glass stopper. FT-IR spectra of PVA-SH and PVA are shown in Figure S1. The absorption at 1056 cm^−1^ corresponds to the characteristic peak of C–O–C in PVA. The absorption at 1409 cm^−1^ is associated with the symmetrical bending of –CH_2_–, while the absorption about 2983 cm^−1^ is attributed to the characteristic peak of –CH_2_– [24]. The broad absorption peak around 3400 cm^−1^ is ascribed to the characteristic peak of the –OH. There is weak absorption about 2532 cm^−1^ attributed to the characteristic vibration absorption of –SH in PVA-SH, respectively [16]. It reveals that the PVA-SH was successfully synthesized.

### 4.3. Preparation of UV-Curable L(-)–Borneol-Functionalized Polyurethane Acrylate Prepolymers (LB-PUAs)

**Figure 10:**
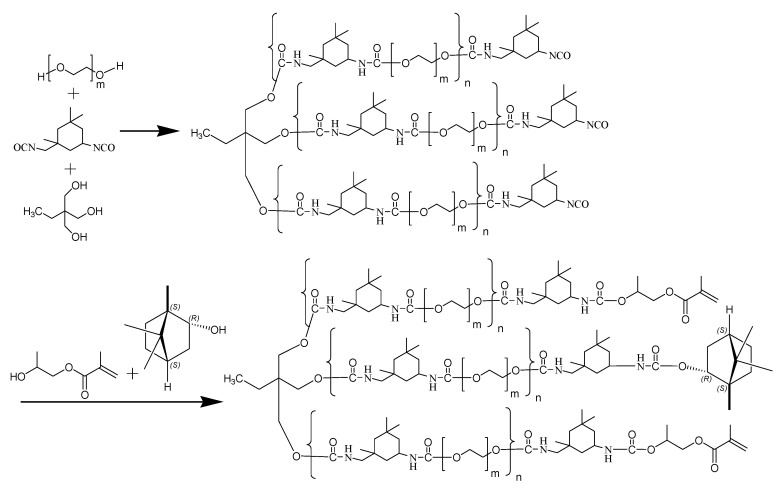
Procedure for fabrication LB-PUAs.

LB-PUAs were prepared according to the procedure presented in Figure 10. If the molar ratio of L(-)–borneol to HPA was higher than 30:70, a large number of L(-)–borneol particles scattered in the LB-PUAs, which indicates that L(-)–borneol is in excess. Therefore, LB-PUAs were prepared by controlling the molar ratio of L(-)–borneol to HPA in the range of 0:100–30:70, as presented in Table 1. According to the formula for preparation of LB-PUAs, after PEG 600, TMP, ditin butyl dilaurate and acetone were added in turn and mixed evenly at 40 °C, IPDI was added slowly for 2 h, and the reaction was conducted at 60 °C for 2 h. After that, a mixture of HPA and L(-)–borneol was introduced into the reaction system at 40°C, and then the mixture was kept at 60 °C for another 4 h. Finally, a transparent sticky liquid of LB-PUAs was produced after the mixture was treated by vacuum distillation at 40 °C for 4 h to remove acetone. The FT-IR and NMR analysis of LB-PUA-20 is presented in Figure S2 and Figure S3, respectively, which reveals the LB-PUAs were successfully produced.

### 4.4. Fabrication of UV-Curable Hydrogels (UV-LBs)

As presented in Figure S4, according to the molar ratio of acrylate groups to thiol groups of 1:1, the homogeneous mixtures of LB-PUAs, PVA-SH and Irgacure-1173 by the formula summarized in Table 2 were poured into the polytetrafluoroethylene molds. Then, the samples were cured using a ZB1000 UV apparatus purchased from Changzhou Zibo Electron Technology Co., Ltd. (the wavelength and light intensity are 365 nm and 10.6 mW cm^−2^, respectively, and the distance of the slides to the UV light is 20 cm) for 20 s each face. The hydrogels produced were named in turn, as shown in Table 2.

### 4.5. Analytical Methods

#### 4.5.1. Fourier-Transform Infrared (FT-IR) Spectroscopic Analysis

FT-IR measurement was carried out with a Nicolet 700 spectrometer (Nicolet Co., Ltd., Madison, WI, USA).

#### 4.5.2. TGA (Thermogravimetric Analysis) Experiment

Thermal stability of UV-curable hydrogels was studied via TGA in a N_2_ atmosphere on a TG 209C apparatus (NETZSCH-Gerätebau GmbH, Selb, Germany). The samples were heated from room temperature to 800 °C, with a heating rate of 10 °C min^−1^.

#### 4.5.3. Swelling Performance

The swelling behavior of the hydrogels (rectangular samples of 25.0 mm × 25.0 mm × 25.0 mm × 1 mm) was averaged after being measured 3 times and calculated using equation swelling rate% = (Wt − W_0_)/W_0_ × 100% [16,17]. Here, W_0_ and W_t_ are the mass of the samples before and after swelling, respectively.

#### 4.5.4. DMA Analysis

The mechanical properties of the UV-curable hydrogels (50 mm × 8 mm × 1 mm) were studied via DMA using a DMA-Q800 instrument (TA Instruments, New Castle, WA, USA) at 25 °C. The load and tensile speed are 0.1 N and 5 mm/min, respectively.

#### 4.5.5. In Vitro Antibacterial Property Study

To study the antibacterial performance, the coated plates and colony count method were conducted 3 times for each sample and averaged [16,17]. The sample without UV-LBs acted as control group (CK). Luria-Bertani (LB) was chosen as the medium for the evaluation of *S*. *aureus* and *E*. *coli*, while YPDA (Yeast Peptone Dextrose Adenine) was chosen for the evaluation of *M*. *albican*. About 20 μL bacterial suspensions (10^5^ CFU·mL^−1^) was introduced into 20 mL media containing UV-LBs (cylindrical samples with diameter and height of 10.0 mm and 1 mm, respectively). After *S. aureus* and *E. coli* were incubated at 37 °C or *M. albican* was incubated at 28 °C for 12 h in a shaking bed under 220 rpm, the antimicrobial activity was calculated using the following equation: bactericidal ratio = OD_CK_ − OD_exp_/OD_CK_ − OD_ini_. Here, OD_CK_, OD_exp_ and OD_ini_ are the values of optical density OD*_600_* recorded on a microplate reader (Infinite 200 Pro, TECAN) for CK, experimental group and initial state, respectively.

#### 4.5.6. Bacterium and Fungi Morphology Study

According to the works reported in [16,17], the bacterial morphology was studied using SEM (SEMS–3000N, Japan, Hitachi Co., Ltd.). The bacterium and fungi co-cultured on the UV-LBs were centrifuged, washed, fixed with 2.5% glutaraldehyde, kept for 24 h at 4 °C, washed with phosphate buffer saline (PBS) and fixed with 1% osmium acid for about 1 h in turn. The bacterial cells were dehydrated with a graded ethanol solution and isoamyl acetate. After the UV-LBs were dried completely and coated with gold–palladium, the bacterial morphology was studied.

#### 4.5.7. In Vitro Cytotoxicity Investigation

The in vitro cytotoxicity was investigated using the optical density of the OD_450_ value against L929 cells according to ref. [15]. The cell viability was obtained according to *Cell viability* (%) = (*OD_s_* − *OD_b_*)/(*OD_n_* − *OD_b_*) × 100%. Here, *OD_s_*, *OD_b_* and *OD_n_* represent the OD_450_ value of the sample investigated, blank group, and negative control group.

#### 4.5.8. Packaging Investigation of Fresh-Cut Banana and Cherry Tomato

The packaging investigation of fresh-cut banana and cherry tomato was conducted, as shown in Figure S5. Slices of fresh-cut cherry tomato and banana were put onto each film of hydrogel and then packaged. To make a comparison, the fresh-cut banana and cherry tomato slices samples exposed to air directly were named CK. After a period of time, the slices of banana and cherry tomato were taken out carefully, and photos were taken by Jizhong Yuan with an iPhone 11 pro to record their status.

#### 4.5.9. Statistical Analysis

The experiment for the antibacterial performance was conducted 3 times, and the means and standard deviations were evaluated with a minimum of 3 experiments using Excel (Microsoft, Redmond, VA, USA).

## Figures and Tables

**Table 1:** Formula for preparation of LB-PUAs.

LB-PUAs	Molar Ratio of L(-)–Borneol to HPA	L(-)–Borneol /mol	HPA /mol	PEG 600/mol	TMP /mol	IPDI /mol
LB-PUA-0	0:100	0	0.0390	0.01	0.002	0.0325
LB-PUA-10	10:90	0.0039	0.0351			
LB-PUA-15	15:85	0.0059	0.0332			
LB-PUA-20	20:80	0.0078	0.0312			
LB-PUA-25	25:75	0.0098	0.0293			
LB-PUA-30	30:70	0.0117	0.0273			

**Table 2:** Formula for preparation of UV-LBs.

UV-LBs	The Categories and Amount of LB-PUAs	The Amount of PVA-SH
UV-LB-0	1 g LB-PUA-0	1.1925 g
UV-LB-10	1 g LB-PUA-10	1.0679 g
UV-LB-15	1 g LB-PUA-15	1.0060 g
UV-LB-20	1 g LB-PUA-20	0.9445 g
UV-LB-25	1 g LB-PUA-25	0.8833 g
UV-LB-30	1 g LB-PUA-30	0.8232 g

The mass of Irgacure-1173 is 3 wt% that of LB-PUAs and PVA-SH.

## Data Availability

The data presented in this study are openly available in the article.

## Supplementary Materials

The following supporting information can be downloaded at: https://www.mdpi.com/article/10.3390/gels12050381/s1, Figure S1: FT-IR spectra of PVA-SH and PVA. Figure S2: FT-IR spectrum of LB-PUA-20. Figure S3: ^1^H-NMR and ^13^C-NMR spectra of LB-PUA-20. Figure S4: Scheme for fabrication UV-LBs. Figure S5: Scheme for the packaging investigation of fresh-cut banana and cherry tomato.

## References

[1] Han P., Sun J.Y., Mao S., Li F.Y., Yan X.X., Zhang T.H., Lu C.W. (2024). Multifunctional pectin-based films containing schiff base triggered by pH microenvironment for freshness monitoring and preservation of fresh-cut papayas. Food Hydrocoll..

[2] Pan X.L., Duan Y., Liu S., Wang Y., Li Q., Jiang F.C., Li Y.X., Huang Z., Su L.J., Li X.B. (2025). All-in-one: Harnessing multifunctional natural polysaccharide spray hydrogel loaded with polyphenol-metal nanoparticles for fruit preservation. Food Chem..

[3] Dwibedi V., Kaur G., George N., Rana P., Ge Y.H., Sun T. (2024). Research progress in the preservation and packaging of fruits and vegetables: From traditional methods to innovative technologies. Food Packag. Shelf Life.

[4] Ganesh K.S., Sridhar A., Vishali S. (2022). Utilization of fruit and vegetable waste to produce value-added products: Conventional utilization and emerging opportunities—A review. Chemosphere.

[5] Han C.Q., Wang W.X., Suo B.X., Zhang J., Ma Q.Y., Sun J.F. (2025). Sodium nitroprusside affects energy metabolism, gamma-aminobutyric acid branching, and circRNA regulation of fresh-cut purple potatoes. Postharvest Biol. Technol..

[6] Qiao L.P., Hou X.R., Li X.K., Hu N.J., Yang X., Wang Y.S., Li X.H., Lu L.F., Liu X. (2025). Glutamate induction of whole potatoes alleviated the browning of fresh cuts: Jasmonate signalling may play a key role. Food Chem..

[7] Liu G.C., Liu R., Fu C.Y., Hu X., Li K., Yan L.K., Yang P., Zhao J. (2025). Protein/polysaccharide composite nanocoating based on amyloid-like aggregation for fresh-cut fruits preservation. Chem. Eng. J..

[8] Liu L.L., Zhang Y.Y., Jin L.X., Abdollahi M., Zhao G.L., Venkatachalam K., Ban Z.J. (2025). Controlled release and stability enhancement of cinnamon essential oil in glutathione-modified soy protein particles: Its antimicrobial application for fresh-cut cantaloupe. Food Res. Int..

[9] Treviño-Garza M.Z., García S., Heredia N., Alanís-Guzmán M.G., Arévalo-Niño K. (2017). Layer-by-layer edible coatings based on mucilages, pullulan and chitosan and its effect on quality and preservation of fresh-cut pineapple (*Ananas comosus*). Postharvest Biol. Technol..

[10] Alessio A., Eugenia G., Casales F.G., Gimenez M.J., Alessandra G., Gugino I.M., Daniela P., Giuseppe S. (2024). Extending shelf-life of fresh-cut apples using manna from ash tree (*Fraxinus angustifolia*) as natural antioxidant agent in comparision with calcium ascorbate. Postharvest Biol. Technol..

[11] Wang D., Li D., Xu Y.Q., Li L., Belwal T., Zhang X.C., Luo Z.S. (2021). Elevated CO_2_ alleviates browning development by modulating metabolisms of membrane lipids, proline, and GABA in fresh-cut Asian pear fruit. Sci. Hortic..

[12] Sharma R., Nath P.C., Das P., Rustagi S., Sharma M., Sridhar N., Hazarika T.K., Rana P., Nayak P.K., Sridhar K. (2024). Essential oil-nanoemulsion based edible coating: Innovative sustainable preservation method for fresh/fresh-cut fruits and vegetables. Food Chem..

[13] Tordi P., Ridi F., Samori P., Bonini M. (2025). Cation-alginate complexes and their hydrogels: A powerful toolkit for the development of next-generation sustainable functional materials. Adv. Funct. Mater..

[14] Rigueto C.V.T., Rosseto M., Alessandretti I., de Oliveira R., Wohlmuth D.A.R., Menezes J.F., Loss R.A., Dettmer A., Pizzutti I.R. (2022). Gelatin films from wastes: A review of production, characterization, and application trends in food preservation and agriculture. Food Res. Int..

[15] Tsai P.-Y., Chen T.-Y., Chuang W.-T., Hsu S.-H. (2025). Self-assembled chitosan-boronic acid hydrogel as dynamic crosslinker to produce 3D-printable glucose-sensitive hydrogel. Carbohydr. Polym..

[16] Wang X.J., Zhu H.Y., Yang Y.N., Lai G.Q., Yang X.F. (2024). UV-curable choline chloride and bromophenol red covalent functionalized chitosan antibacterial and pH-sensitive hydrogels. Food Hydrocoll..

[17] Wang X.J., Yuan J.Z., Sun N.N., Jiang Y.H., Yu Y.C., Lai G.Q., Yang X.F. (2025). UV-Curable antibacterial and pH-sensitive eugenol functionalized chitosan-polyurethane hydrogels for shelf-life extension of chicken. Food Control.

[18] Mao L., Wang C.Y., Dong Z.Y., Yao J., Dong F., Dai X.L. (2025). Fabrication of polylactic acid bilayer composite films using polyvinyl alcohol based coatings containing functionalized carbon dots and layered clay for active food packaging. Ind. Crops Prod..

[19] Wu J.J., Zhang Y., Zhang F.Y., Mi S., Yu W.L., Sang Y.X., Wang X.H. (2025). Preparation of chitosan/polyvinyl alcohol antibacterial indicator composite film loaded with AgNPs and purple sweet potato anthocyanins and its application in strawberry preservation. Food Chem..

[20] Sheng W.Y., Yang L., Yang Y.C., Wang C.Z., Jiang G.Y., Tian Y.Q. (2025). Photo-responsive Cu-tannic acid nanoparticle-mediated antibacterial film for efficient preservation of strawberries. Food Chem..

[21] Zhu Y.D., Pang X.H., Zhang W.L., Zhang C., Zhang B.L., Fu J.M., Zhao H.F., Han W.J. (2025). Green synthesis of silver nanoparticles using persimmon polysaccharides for enhanced polysaccharide-based film performance. Food Res. Int..

[22] Wang Q., Zhang Z., Yin J., Shen L.Y., Zhu L.L., Redshaw C., Zhang Q.L. (2025). Block cationic copolymer/quaternary ammonium chitosan-based composite antibacterial hydrogel dressings with NIR photothermal effects for bacteria-infected wound healing. Int. J. Biol. Macromol..

[23] Stancu A.I., Ditu L.M., Oprea E., Ficai A., Badea I.A., Buleandra M., Brîncoveanu O., Mirea A.G., Voicu S.N., Musuc A.M. (2025). New antimicrobial gels based on clove essential oil-cyclodextrin complex and plant extracts for topical use. Gels.

[24] Galaburri G., Infantes-Molina A., Queirolo C.M.M., Mebert A., Tuttolomondo M.V., Rodríguez-Castellón E., Lázaro-Martínez J.M. (2025). Composite films based on linear polyethyleneimine polymer and starch or polysaccharides from DDGS: Synthesis, characterization, and antimicrobial studies. Polymers.

[25] Somwongin S., Tammasorn P., Limbunjerd R., Norkaew K., Lertprachyakorn N., Kongsaeng T., Phokasem P., Disayathanoowat T., Lin W.C., Chaiyana W. (2025). Bee product-based antimicrobial film-forming gels targeting *Staphylococcus aureus*, *Staphylococcus epidermidis*, and *Cutibacterium acnes* for anti-acne applications. Gels.

[26] Cai Z.H., Wang L.Y., Zhang Q.Q., Yang W.Y., Zhang C., Wang H., Xiao H.M. (2025). Eco-friendly coating engineered with antimicrobial lipopeptides maintains freshness and induces genes expression in anthocyanin biosynthesis of blueberry. Int. J. Biol. Macromol..

[27] Li C.H., Zhao Y.X., Zhang A.J., Xu Y.C., Wang H.Y. (2025). Preparation, characterization, and antibacterial properties of a soybean protein isolate/gelatin composite film containing rosemary-modified bentonite and application of fresh lemon slices. Int. J. Biol. Macromol..

[28] Kumar B.T.S., Reddy J.P., Vanajakshi V., Dasalkar A.H., Yannam S.K., Hebbar U.H., Singh S.A. (2025). Development and characterization of carrageenan-based antibacterial films incorporated with natural melanin pigment from niger seed hulls (*Guizotia abyssinica*) and their efficacy to enhance the shelf-life of strawberries. Food Control.

[29] Lu Z.W., Mu J.B., Guan C.W., Sui T.S., Liu C.Z., Guo Z.C., Liao S.M. (2025). Green and recyclable photocatalytic hydrogel film with antibacterial and ethylene scavenging properties for fruit preservation. Food Chem..

[30] Yang H.J., Li L.P., Li C., Xu Z.H., Tao Y.H., Lu J., Xia X.D., Tan M.Q., Du J., Wang H.S. (2024). Multifunctional and antimicrobial carboxymethyl cellulose-based active hydrogel film for fruits packaging and preservation. Food Biosci..

[31] Yu K.J., Zhang S.Y., Yang L.N., Liu H., Li X.P., Xu Y.X., Li J.R. (2025). Strong, tough, antibacterial, antioxidant, biodegradable multi-functional intelligent hydrogel film for real-time detection and maintenance of salmon freshness. Food Res. Int..

[32] Gong W., Yang T.Q., He W.Y., Li Y.X., Hu J.N. (2025). On-demand removable hydrogel film derived from gallic acid-phycocyanin and polyvinyl alcohol for fruit preservation. Food Chem..

[33] Wu Y.B., Gu Z.M., Chen T.T., Zu D.T., Gan Y.H., Chen H.L., Yang J.N., Yu X., Cai H.H., Sun P.H. (2025). Effect of different crosslinking agents on carboxymethyl chitosan-glycyrrhizic acid hydrogel: Characterization and biological activities comparison. Int. J. Biol. Macromol..

[34] Qian Y., Zheng Y., Jin J., Wu X., Xu K., Dai M., Niu Q., Zheng H., He X., Shen J. (2022). Immunoregulation in diabetic wound repair with a photoenhanced glycyrrhizic acid hydrogel scaffold. Adv. Mater..

[35] Laquerbe S., Sayed J.E., Lorthioir C., Meyer C., Narita T., Ducouret G., Perrin P., Sanson N. (2023). Supramolecular crosslinked hydrogels: Similarities and differences with chemically crosslinked hydrogels. Macromolecules.

[36] Mehta P., Sharma M., Devi M. (2023). Hydrogels: An overview of its classifications, properties, and applications. J. Mech. Behav. Biomed. Mater..

[37] Wang D.B., Zhu C.Q., Yang Q.F., Xu Y.Q., Zhang D.Q., Wang D.Y., Liu F., Hou C.L. (2025). Stretchable, controlled release of active substances, and biodegradable chitosan-polyvinyl alcohol hydrogel film for antibacterial and chilled meat preservation. Food Chem..

[38] Sanchez-Cid P., Alonso-Gonzalez M., Jimenez-Rosado M., Benhnia M.R.E., RuizMateos E., Ostos F.J., Romero A., Perez-Puyana V.M. (2024). Effect of different crosslinking agents on hybrid chitosan/collagen hydrogels for potential tissue engineering applications. Int. J. Biol. Macromol..

[39] Dodda J.M., Azar M.G., Sadiku R. (2021). Crosslinking trends in multicomponent hydrogels for biomedical applications. Macromol. Biosci..

[40] Lechuga-Islas V.D., Gillissen E., Bourguignon M., Grignard B., Detrembleur C. (2025). Foam-to-adhesive recycling of self-blown non-isocyanate polyurethane foams facilitated by integration of disulfide exchangeable bonds and moisture. Chem. Eng. J..

[41] Wang W., Coenye T., Su J.Y., Qiu S.-X. (2025). Biofilm inhibition and eradication activity of citral and borneol against foodborne bacteria. Ind. Crops Prod..

[42] Zhou Z.L., Chen R.X., Li P.Z., Fan P.H., Ma L., Cai X.Y., Hou Y.C., Li B.B., Su J.Y. (2025). Natural borneol improves cellular uptake of curcumin to enhance its photodynamic bactericidal activity against Escherichia coli ATCC 8739. Food Microbiol..

[43] Zhao Z.J., Fan X., Li X.Y., Qiu Y.W., Yi Y.F., Wei Y.P., Wang Y. (2024). All-natural injectable antibacterial hydrogel enabled by chitosan and borneol. Biomacromolecules.

[44] Gao X., Zhang H.C., Yan C.F., Wu J., Wang Y.T., Jiang M.H., Wang Y.N. (2025). Yunnan Baiyao-enhanced cellulose nanofiber composite hydrogel wearable patch for transdermal drug delivery and anti-freezing applications. Int. J. Biol. Macromol..

[45] Deng Z.X., Guo Y., Wang X.F., Song J.J., Yang G., Shen L.T., Wang Y.H., Zhao X., Guo B.L., Wang W. (2025). Multiple crosslinked, self-healing, and shape-adaptable hydrogel laden with pain-relieving chitosan@borneol nanoparticles for infected burn wound healing. Theranostics.

[46] Liu Z.Z., Liao H.B., Li H.L., Zou Z.R. (2025). Fabrication and characterization of *Cinnamomum camphora* chvar. *Borneol* essential oil microcapsules decorated by β-cyclodextrin with ultrasound-assisted complexation method. Arab. J. Sci. Eng..

[47] Abbasi Z., Esfandiari Z., Rostamabadi H. (2025). Postbiotic-loaded κ-carrageenan hydrogels double cross-linked with carboxymethyl cellulose and calcium ions. Food Hydrocoll..

[48] Yang Z.M., Liu Y., Wang E.Z., Yin W., Wang Y.J., Guo Y.C., Zhang W.T., Qi H. (2026). Insights into the highly selective and efficient adsorption of Pb^2+^ by fish skin collagen-enabled sodium alginate-based composite gel spheres: Adsorption and interference mechanisms. Food Hydrocoll..

[49] Feng X., Li M., Li S.H., Lin M.T., Nie Y., Yao N., Deng T.X., Yang X.H., Ding H.Y., Xu L.N. (2024). Fabrication and properties of recyclable tung oil-based polymer coatings based on dual cross-linked dynamic covalent polymer networks. Prog. Org. Coat..

[50] Kim G.Y., Sung S.J., Kim M.P., Kim S.C., Lee S.H., Park Y.I., Noh S.M., Cheong I.W., Kim J.C. (2020). Reversible polymer networks based on the dynamic hindered urea bond for scratch healing in automotive clearcoats. Appl. Surf. Sci..

[51] Liu W.Z., Li L., Liu S.N., Liu B., Wu Z.Y., Deng J.R. (2024). Novel robust ion-specific responsive photonic hydrogel elastomer. J. Mater. Chem. C.

