# Supplementary material for: UV-Curable L(-)–Borneol-Functionalized Antibacterial Hydrogels for Packaging of Fresh-Cut Banana and Cherry Tomato
Source: Gels. 2026 Apr 30;12(5):381. doi: 10.3390/gels12050381 (PMC13205466; doi:10.3390/gels12050381)
Supplement: Supplementary file 1 [file gels-12-00381-s001.zip › gels-4261375-supplementary.pdf]

Supplementary Materials for

UV-Curable L(-)–Borneol-Functionalized Antibacterial Hydrogels for Packaging of Fresh-Cut Banana and Cherry Tomato

Jizhong Yuan [1,†], Yaohuang Jiang [2,†], Mengle Liu [1], Peipei Wu [1], Guoxian Feng [1], Yanchun Yu [2,*] and Xiongfa Yang [1,*]

([1]Key Laboratory of Organosilicon Chemistry and Material Technology, Ministry of Education, Zhejiang Key Laboratory of Organosilicon Material Technology, College of Material, Chemistry and Chemical Engineering, Hangzhou Normal University, Hangzhou, 311121, Zhejiang, China

[2] College of Life and Environmental Sciences, Hangzhou Normal University, Zhejiang, 311121, China)

*Authors for the correspondence: Yanchun Yu, Email address: ycyu@hznu.edu.cn. Xiongfa Yang, Email address: yangxiongfa@hznu.edu.cn.

Number of Pages: 6

Number of Figures: 5

1 FT-IR spectra of PVA-SH and PVA

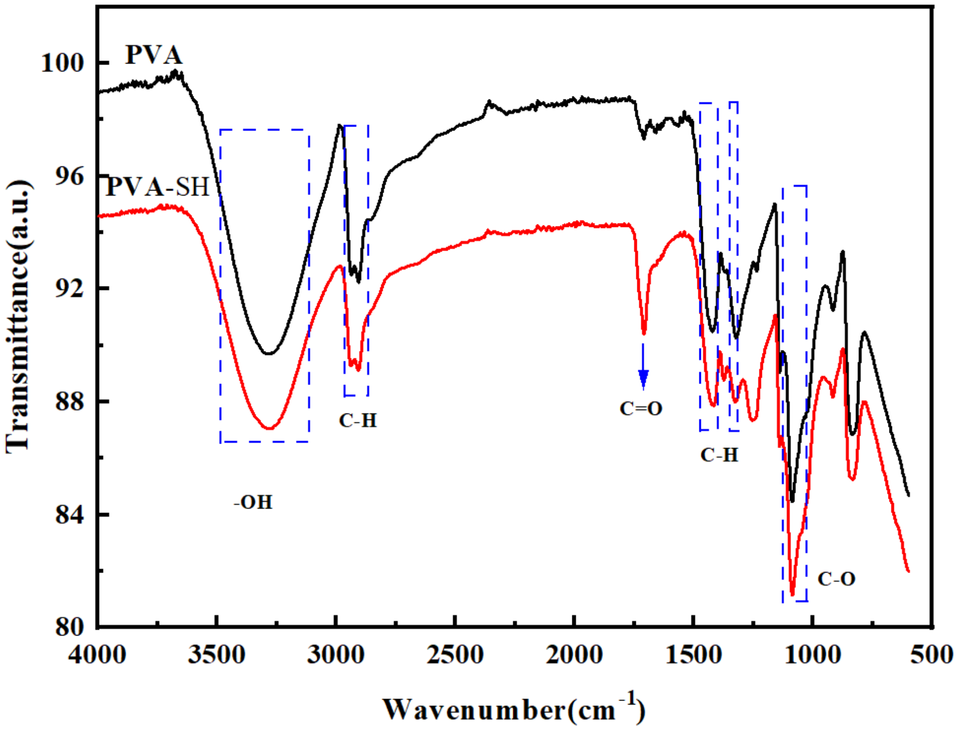

(a)FT-IR spectra of PVA-SH and PVA over 4000-500 cm^{-1}

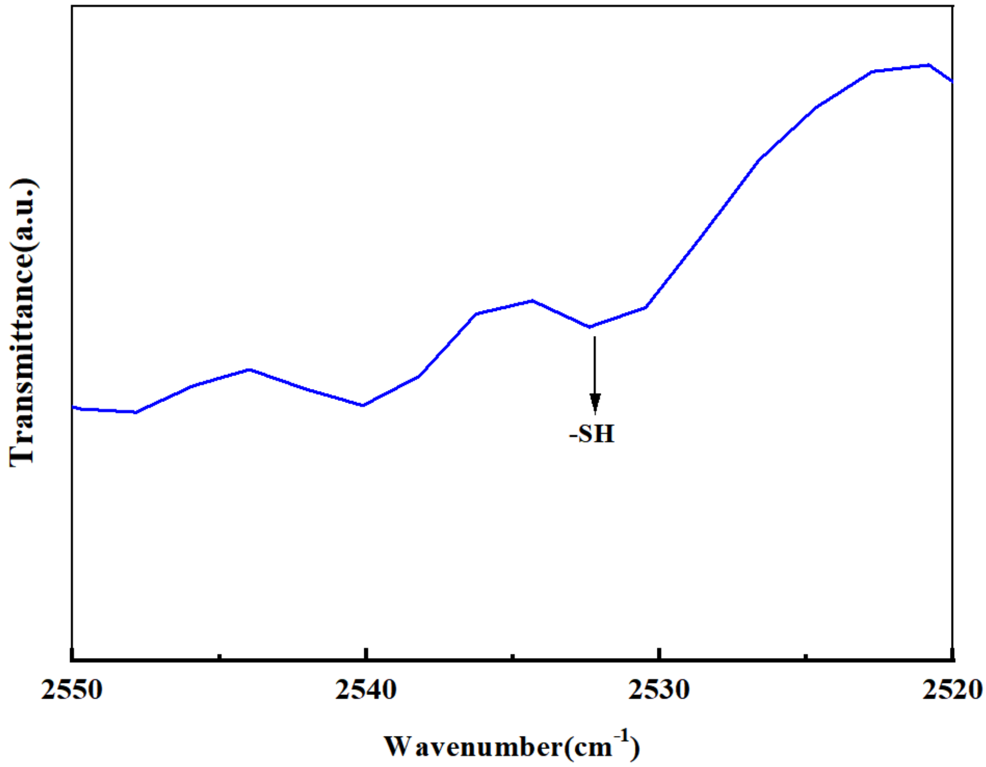

(b)FT-IR spectrum of PVA-SH over 2550–2520 cm^{-1}

Figure **S1** FT-IR spectra of PVA-SH and PVA

The absorption at 1056 cm^{-1} corresponds to the symmetric stretching vibration of C–O–C in PVA. The absorption at 1409 cm^{-1} is associated with the symmetrical bending of –CH_2– while the absorption about 2983 cm^{-1} is attributed to the asymmetric and symmetric stretching vibrations of –CH_2– [S1]. The broad absorption peak around 3400 cm^{-1} is ascribed to the stretching vibrations of the –OH. There is a weak absorption about 2532 cm^{-1} ascribed to the characteristic vibration absorption of –SH in PVA-SH, respectively [S2].

2 FT-IR analysis of LB-PUA-20

The LB-PUA-20 was analyzed by FT-IR as exhibited in Fig. S2. The absorption at 3458–3210 cm^{-1} was assigned to the stretching vibrations of –OH and NH_2. The absorption at 2956–2870 cm^{-1} was ascribed to the stretching vibration of –C–H. The absorptions at 1699–1703 cm^{-1} and 1418–1420 cm^{-1} were attributed to the stretching vibration of –C=O and deformation vibration of –CH_3 in –$NHCOCH_3$ groups, respectively. There was a weak characteristic absorption of –C=C– in acrylate groups at 1620 cm^{-1}. It indicates that LB-PUAs were prepared successfully.

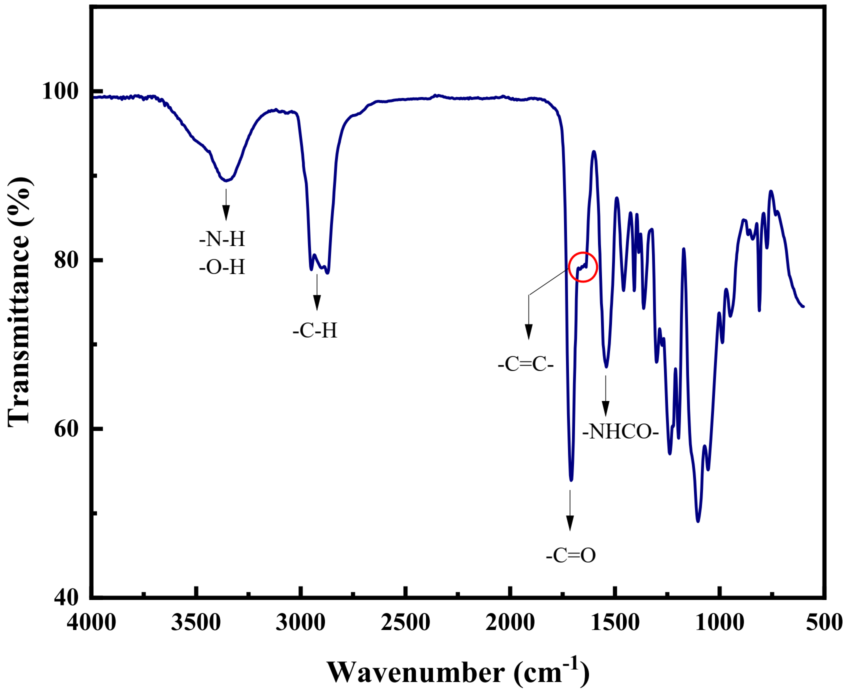

Figure **S2**. FT-IR spectrum of LB-PUA-20

3 ^{1}H-NMR and ^{13}C-NMR analysis of LB-PUA-20

^{1}H-NMR and ^{13}C-NMR analysis of LB-PUA-20 were conducted as shown in Fig. S3.

^{1}H-NMR (Fig. S3a): The chemical shifts in the range of 5.68–6.47 ppm and 2.62–3.04 ppm were ascribed to the protons in $–OCN\mathit{H}CH_2–$ and $–OCNHC\mathit{H}_2–$, respectively. The chemical shift at 3.44–4.32 ppm was ascribed to the protons of $–OC\mathit{H}_2C\mathit{H}_2O–$ in PEG 600 units. The chemical shifts at 1.23–0.92 ppm was ascribed to the protons of $–C\mathit{H}_3$ in the IPDI units. The chemical shifts at 1.16 ppm and 1.42 ppm were ascribed to the protons of $–C\mathit{H}_3$ in the L(-)borneol groups, while the chemical shifts at 1.24 ppm, 1.27 ppm, 1.49 ppm, 1.52 ppm and 1.76 ppm were ascribed to the protons of $−CH_2−$ in the LB groups.

^{13}C-NMR (Fig. S3b): The chemical shifts in the range of 145–158 ppm was ascribed to the carbon atoms in –C=O of $–O\mathit{C}NHCH_2–$. The chemical shifts about 70 ppm were ascribed to the carbon atoms of $–OCH_2CH_2O–$ in PEG 600 units. The chemical shifts at 19–30 ppm and 30–58 ppm were ascribed to the carbon atoms $\mathit{C}(CH_3)_2$ and $–\mathit{C}H_3$ in the L(-)borneol groups, respectively.

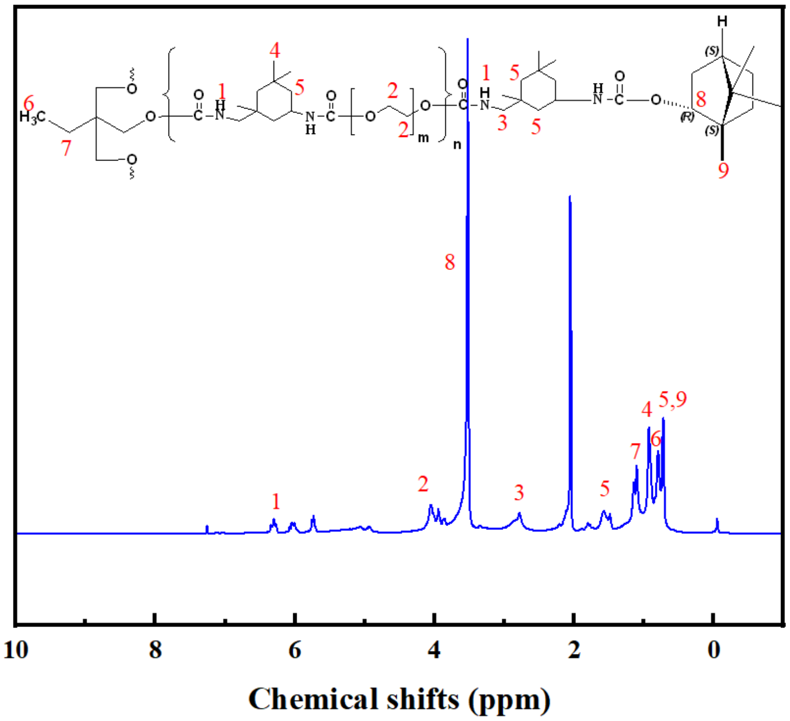

(a) ^{1}H-NMR spectrum

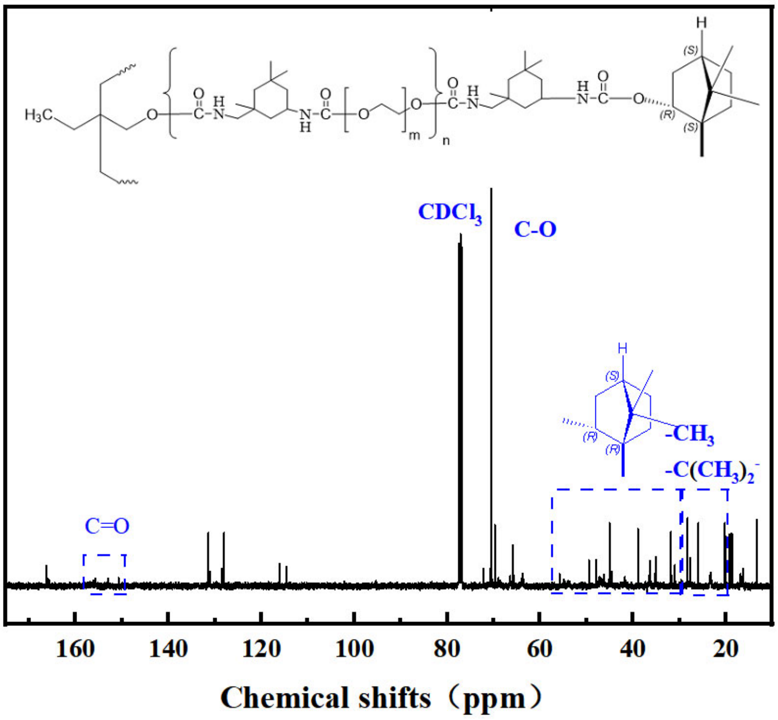

(b) ^{13}C-NMR spectrum

Figure **S3**. ^{1}H-NMR and ^{13}C-NMR spectra of LB-PUA-20

4 Scheme for fabrication UV-LBs

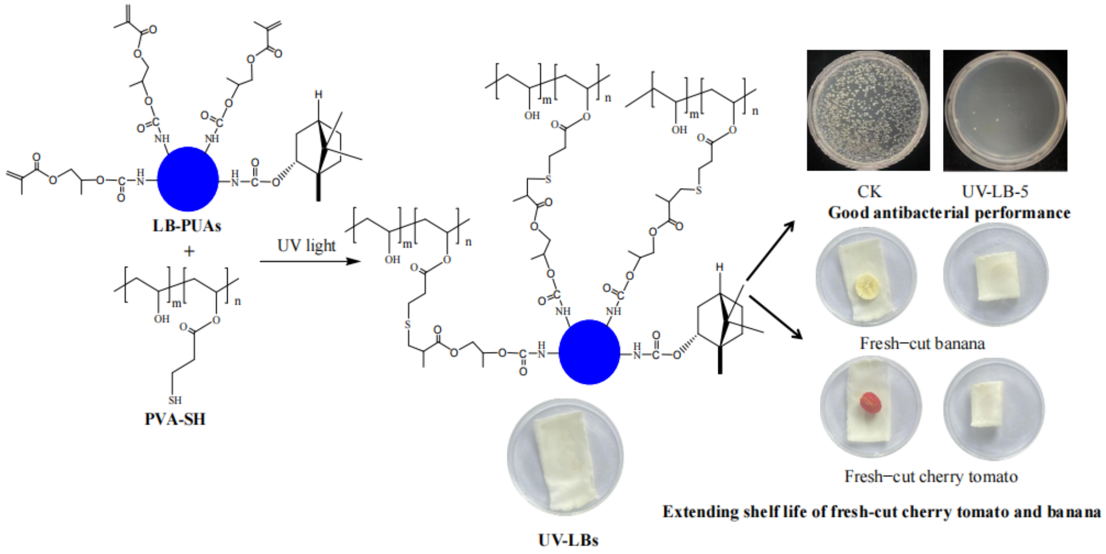

Figure **S4**. Scheme for fabrication UV-LBs

5 Procedure for the packaging investigation of fresh-cut banana and cherry tomato

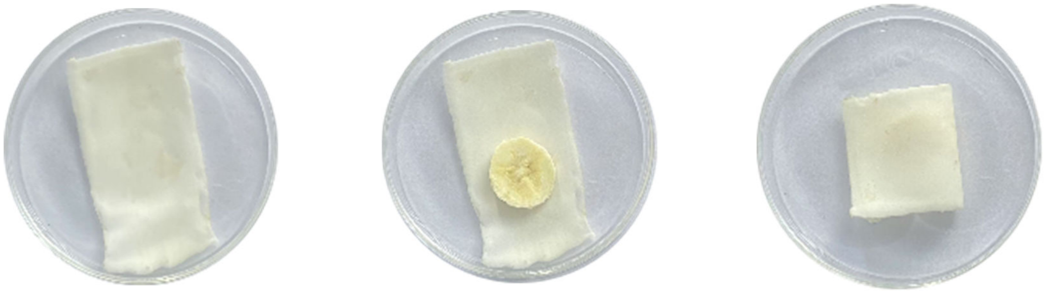

Hydrogel | Fresh-cut banana on hydrogel | Fresh-cut banana packaged by hydrogel

(a) Fresh-cut banana

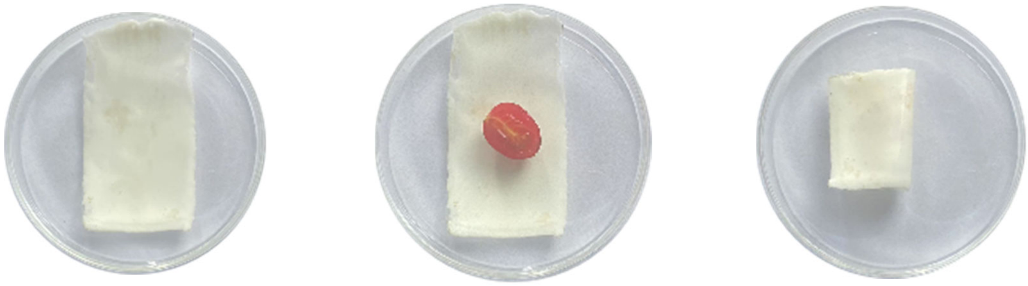

Hydrogel | Fresh-cut cherry tomato on hydrogel | Fresh-cut cherry tomato packaged by hydrogel

(b) Fresh-cut cherry tomato

Figure S5. Scheme for the packaging investigation of fresh-cut banana and cherry tomato

References

S1. Lu Z. W., Mu J. B., Guan C. W., Sui T. S., Liu C. Z., Guo Z. C., Liao S. M. Green and recyclable photocatalytic hydrogel film with antibacterial and ethylene scavenging properties for fruit preservation. *Food Chemistry*, 2025, 475, 143266.

S2. Wang X. J., Zhu H. Y., Yang Y. N., Lai G. Q., Yang X. F. UV-curable choline chloride and bromophenol red covalent functionalized chitosan antibacterial and pH-sensitive hydrogels. *Food Hydrocolloids*, 2024, 154, 110103.
